# Seroprevalence of antibodies against hepatitis A and E among the general population in 5 provinces, Lao People’s Democratic Republic: Variation according to location

**DOI:** 10.1371/journal.pone.0329384

**Published:** 2025-08-14

**Authors:** Vilaysone Khounvisith, Siriphone Virachith, Nouna Innoula, Latdavone Khenkha, Bounta Vongphachanh, Jan Hattendoft, Peter Odermatt, Judith M Hübschen, Antony P Black

**Affiliations:** 1 LaoLuxLab/Vaccine Preventable Diseases Laboratory, Institut Pasteur du Laos, Vientiane, Lao PDR; 2 Swiss Tropical and Public Health Institute, Allschwil, Switzerland; 3 University of Basel, Basel, Switzerland; 4 Department of Infection and Immunity, Luxembourg Institute of Health, Esch-sur-Alzette, Grand-Duchy of Luxembourg; 5 School of Life Sciences, College of Liberal Arts and Sciences, University of Westminster, London, United Kingdom; CEA, FRANCE

## Abstract

Hepatitis A and E viruses (HAV and HEV) are transmitted through the faecal-oral route: via contaminated food, water, and contact with infected people and/or animals for HEV. Due to limited data from Lao People’s Democratic Republic (Lao PDR), we assessed HAV and HEV seroprevalence in the Lao general population. A cross-sectional study collected 2412 serum samples and demographic information from participants (5–93 years) across five provinces. Anti-HAV (IgM and IgG) and anti-HEV antibodies (IgG) were detected by enzyme-linked immunosorbent assay (Dia.Pro). The overall seroprevalence of anti-HAV was 84.3% and anti-HEV was 57.9%. Seropositivity was associated with occupation, location, increasing age, ethnicity (only for anti-HAV) and sex (only for anti-HEV). The age at which 50% of the population was seropositive differed from 12 years (Oudomxay) to 26 years (Savannakhet and Vientiane) for anti-HAV and from 22 years (Savannakhet) to 49 years (Vientiane) for anti-HEV. The prevalence of double seropositivity was high overall (53.4%), particularly in Savannakhet and Champasack. These significant differences according to location and socio-demographics may be the result of variation of exposure to the viruses, such as through water, sanitation and hygiene-related risks, occupational exposure and animal contact. Further studies are warranted to identify the most important risks for transmission in Lao PDR in order to develop targeted public health interventions.

## Introduction

Hepatitis A virus (HAV) is a common cause of acute viral hepatitis worldwide. The transmission route is faecal-oral through contact with infected people, and contaminated food or water [1]. In 2019, the Global burden of disease data estimated that HAV infected 159 million people globally, leading to 39000 deaths and 2.3 million disability-adjusted life years [2]. In low- and middle-income countries, cases are commonly associated with poor water, sanitation and hygiene (WASH) conditions [2,3]. HAV infection is usually asymptomatic and self-limiting in young children, but in adulthood, symptoms occur more frequently. It is thought that anti-HAV IgG induced following infection may lead to lifelong protection [2]. Vaccines against HAV are available, but their use depends on the local epidemiological context. Thus, for example, in highly endemic countries, routine HAV vaccination is not recommended as it may shift the burden of infection from children to adolescents and adults and therefore increase the number of symptomatic cases [2].

Hepatitis E virus (HEV) is a common infection in low- and middle-income countries [4,5]. HEV is divided into 8 genotypes. HEV genotypes 1 and 2 are transmitted by the faecal-oral route, through poor hygiene and unsafe drinking water, for instance, whereas genotypes 3,4 and 7 are zoonotic diseases infecting humans, swine and other animals [6]. Recently identified genotypes 5,6 and 8 are thought to only infect animals [7]. The World Health Organisation (WHO) estimated that 20 million people are infected with HEV every year with approximately 3.3 million symptomatic cases worldwide [8]. Rarely, acute hepatitis E can lead to severe fulminant hepatitis and acute liver failure, risking death. Infection during pregnancy, especially in the second or third trimester, is associated with a higher risk for acute liver failure, foetal loss and mortality and in the third trimester with a 15–20% fatality rate for pregnant women [9]. The HEV vaccine is not widely available outside of China [8]. As with anti-HAV, the anti-HEV antibodies are long-lived, and seroprevalence can be used as reliable marker for previous exposure.

Lao People’s Democratic Republic (PDR) is a landlocked country in Southeast Asia, divided in 17 provinces plus one prefecture. Our previous study suggested high exposure to HAV in the Lao general population with an IgG seroprevalence of 62% in Xiengkhouang province and 45.5% in Vientiane capital [10] as well as 63.2% in rural Khammouane province (manuscript in preparation).

In addition, Lao PDR has high circulation of HEV infection. In our previous studies, anti-HEV seroprevalence was 41.0% in swine contacts and 18.1% in the control group, in Vientiane capital [11] and 43.9% −57.7% in the general population, with risk factors such as being male, having close contact with cattle and eating undercooked meat [12 and manuscript in preparation].

Although we have previously reported seroprevalence of anti-HAV and anti-HEV antibodies, the studies focused on a limited area with a limited sample size, and the age-stratified seroprevalence in the Lao general population in different regions is not well documented. Therefore, this study aimed to determine the prevalence of anti-HAV and anti-HEV antibodies in the general population in 5 provinces in Lao PDR to estimate the burden and to provide recommendations about prevention.

## Methodology

### Participants and recruitment

This study made use of 2412 serum samples collected from August to September 2020 in the context of a cross-sectional anti-SARS-CoV-2 sero-study. Details of the method were described previously [13]. In brief, healthy participants were randomly selected by using cluster sampling design to select households in three regions: Vientiane Capital, the North (Oudomxay and Luangprabang), and the South (Champasack and Savannakhet), targeting 900 participants in each region. In the Northern and Southern regions, two provinces were selected, each with a sample size of 450 participants per province. In each province, random selection was used to select 3 districts per province and then 6 villages and 5 households of each village were randomly selected. All inhabitants aged five and above in each household were invited to participate. If a target household could not be reached due to difficulty of access, the nearest household in that village was selected as a replacement. The objectives of the study were explained to the participants and informed consent was obtained prior to their participation. Following this, a structured questionnaire was administered to collect data on socio-demographic characteristics and 5 ml of blood was collected into dry collection tubes. Serum was separated by centrifugation and stored at 4°C for a short period and transferred to Institut Pasteur du Laos for long-term storage at −80°C until testing.

### Antibodies detection

Anti-HAV (IgM and IgG) and anti-HEV (IgG only) antibodies were detected by commercial enzyme-linked immunosorbent assay (ELISA) according to the manufacturer’s instructions (Dia.pro, Milan, Italy). Cut-off OD values were determined using the formula: (negative control + positive control)/3 and samples with a value of greater than 1.1 were considered positive, lower than 0.9 as negative and 0.9–1.1 as borderline and grouped with the negative results for analysis. The sensitivity and specificity of the tests were 100% and 98% for anti-HAV and 100% and 100% for anti-HEV, respectively.

### Statistical analysis

The data were analysed using Microsoft Excel and STATA version 14. The frequencies for categorical variables were described. The seroprevalence of anti-HAV and anti-HEV was determined as percentage and 95% confidence intervals (CI). Bivariate analyses were used to determine the association between the independent and outcome variables (anti-HAV and anti-HEV) and multivariable analyses were used in the final model, which we included: age, gender, occupation, provinces and ethnicity. Due to limited numbers of participants, for multivariable analysis, ages were grouped into those 20 years old or less and those aged more than 20 years old. However, as it is important to compare seroprevalence between locations at all ages, we used logistic regression with generalized estimating equations to account for the clustering of the data within province to calculate the predicted prevalence across ages. The age at which half of the population were anti-HAV and anti-HEV seropositive was estimated for each province by using the marginal effects.

### Ethics approval

This study was approved by the Lao National Ethics Committee for Health Research (NECHR) (Ref #052/2020).

## Results

### Socio-demographics

Between August and September 2020, 2412 healthy participants were recruited into this cross-sectional serostudy, to determine the age-stratified seroprevalence of anti-HAV and anti-HEV antibodies (exposure). Six individuals refused to participate due to fear of needles and reluctance to answer questions. More than half of the participants were female (58.1%). The mean age of the population was 42.7 ± 16.8 years (age range; 5–93 years) and more than 30% of the participants came from Vientiane capital. Farmer/labour worker was the main occupation (917/2410; 38.1%) and Lao Loum was the principal ethnicity of participants (1892/2412; 78.4%) (Table 1).

**Table 1:** Socio-demographic characteristics of study participants.

Characteristics	N = 2412	Percentage
**Sex**		
* Male*	1011	41.9
* Female*	1401	58.1
**Age (years)**		
* ≤ 10*	51	2.1
* 11-20*	244	10.1
* 21-30*	318	13.2
* 31-40*	457	18.9
* 41-50*	520	21.5
* 51-60*	471	19.5
* > 60*	351	14.6
**Province**		
* Vientiane capital*	730	30.3
* Champasack*	414	17.2
* Luangprabang*	461	19.2
* Oudomxay*	396	16.4
* Savannakhet*	411	17.0
**Occupation, n = 2410**		
* Student*	231	9.6
* Unemployed*	675	28.0
* Farmer/Labour worker*	917	38.1
* Commerce/business*	371	15.4
* Office staff*	216	9.0
**Ethnicity**		
* Lao-Loum*	1892	78.4
* Hmong-Mien*	80	3.3
* Chinese-Tibetan*	20	0.8
* Mon-Khmer*	417	17.3
* Other/NA*	3	0.1

### Seroprevalence of anti-HAV

**Fig 1:**
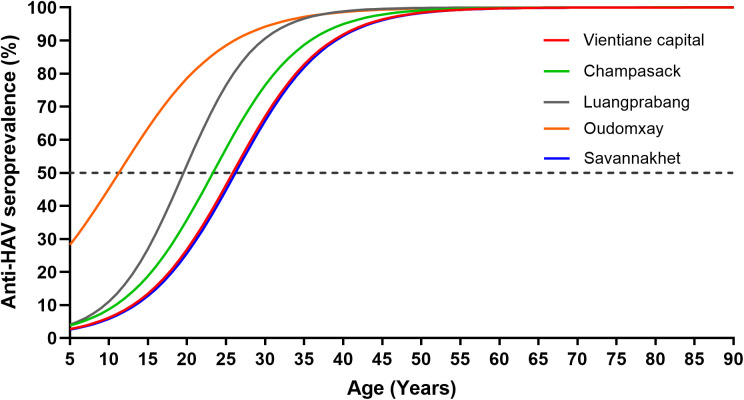
Anti-HAV seroprevalence according to province. The seroprevalence curves were generated by generalised estimating equations. Dotted line represents the 50% seroprevalence level.

Of the 2412 participants, 2034 (84.3%) were seropositive for anti-HAV. The anti-HAV seroprevalence increased from 6/51 (11.8%) in those aged up to 10 years to 349/351 (99.4%) in those aged >60 and from 77.1% in Vientiane capital to 92.9% in Oudomxay province. The age at which 50% of the study population were estimated to be seropositive was around 12 years in Oudomxay province, compared to 26 years in Vientiane capital and Savannakhet (Fig 1).

The anti-HAV seropositivity in the older age group (>20 years old) was significantly higher than that in the younger age group (aOR=19.8, 95% CI = 12.5–31.4, p < 0.001). Additionally, non-Lao-Loum individuals had a statistically higher seroprevalence compared to Lao-Loum participants (aOR=2.9, 95% CI = 1.5–5.4, p = 0.001). Students demonstrated a lower seroprevalence compared to all other occupations. Furthermore, participants from Luangprabang (aOR=2.1, 95% CI = 1.3–3.5, p = 0.002), and Oudomxay provinces (aOR=2.4, 95% CI = 1.2–5.1, p = 0.013) had statistically higher seroprevalence rates than Vientiane capital. A similar percentage of male and female participants were anti-HAV seropositive (Table 2).

**Table 2:** Multivariable analysis of factors associated with anti-HAV seropositivity among 2412 participants.

Characteristic	Overall	Positive	Bivariate analysis		Multivariable analysis	
	*N = 2412*	*N = 2034*	OR (95% CI)^*1*^	p-value	aOR (95% CI)^*2*^	p-value
**Age in years, n (%)**						
* ≤ 20*	295	79 (26.8)	Ref		Ref	
* > 20*	2117	1955 (92.4)	33.0 (24.4–44.7)	0.011	19.8 (12.5–31.4)	<0.001
**Sex, n (%)**						
* Male*	1011	846 (83.7)	Ref		Ref	
* Female*	1401	1188 (84.8)	1.1 (0.9 - 1.4)	0.457	0.8 (0.6–1.1)	0.189
**Ethnicity, n (%)**						
* Lao-Loum*	1892	1558 (82.4)	Ref		Ref	
* Non-Lao-Loum*	520	476 (91.5)	2.3 (1.7 - 3.3)	<0.001	2.9 (1.5–5.4)	0.001
Occupation, n (%) *						
* Student*	231	52 (22.5)	Ref		Ref	
* Business/commerce*	371	331 (89.2)	28.5 (18.2 - 44.7)	<0.001	3.8 (2.1–7.0)	<0.001
* Farmer/Labour worker*	917	829 (90.4)	32.4 (22.2 - 47.4)	<0.001	3.6 (2.1–6.1)	<0.001
* Office staff*	216	199 (92.1)	40.3 (22.5 - 72.2)	<0.001	4.8 (2.3–9.9)	<0.001
* Unemployed*	675	621 (92.0)	39.5 (26.1 - 60.0)	<0.001	6.7 (3.9–11.5)	<0.001
**Province, n (%)**						
* Vientiane capital*	730	563 (77.1)	Ref		Ref	
* Champasack*	414	353 (85.3)	1.72 (1.3 - 2.4)	0.001	1.5 (0.9–2.3)	0.056
* Luangprabang*	461	409 (88.7)	2.33 (1.7 - 3.3)	<0.001	2.1 (1.3–3.5)	0.002
* Oudomxay*	396	367 (92.9)	3.89 (2.6 - 6.0)	<0.001	2.4 (1.2–5.1)	0.013
* Savannakhet*	411	342 (83.4)	1.49 (1.1 - 2.1)	0.015	1.1 (0.7 −1.6)	0.7

Age-groups were collated to allow greater power of analysis.

^1^OR, odds ratio; CI, confidence interval.

^2^aOR, adjusted odds ratio; CI, confidence interval.

*Occupation, n = 2410.

### Seroprevalence of anti-HEV

**Fig 2:**
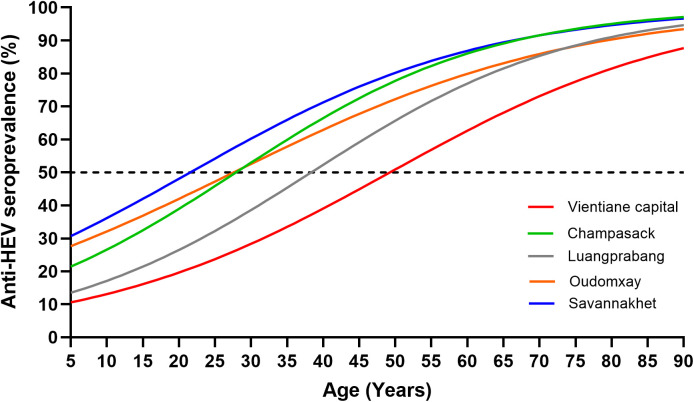
Anti-HEV seroprevalence according to province. The seroprevalence curves were generated by generalised estimating equations. Dotted line represents the 50% seroprevalence level.

Of the 2412 participants, 1396 (57.9%) were seropositive for anti-HEV (IgG). The anti-HEV seroprevalence increased from 2/51 (3.9%) in those aged up to 10 years to 273/351 (77.8%) in those aged >60 and from 43.2% in Vientiane capital to 73.7% in Savannakhet. The age at which 50% of the study population were estimated to be seropositive was around 22 years in Savannakhet province compared to 49 years in Vientiane capital (Fig 2).

As with anti-HAV, the anti-HEV seropositivity in the older age group (>20 years old) was significantly higher than that in the younger age group (aOR=4.6, 95% CI = 3.0–7.0, p < 0.001). Females showed a lower seroprevalence than males (aOR=0.4, 95% CI = 0.4–0.5, p=<0.001). Students demonstrated a lower seroprevalence compared to other occupations. Furthermore, participants from Vientiane capital had significantly lower seroprevalence than all other provinces (Table 3).

**Table 3:** Multivariable- analysis of factors associated with anti-HEV seropositivity among 2412 participants.

Characteristic	Overall	Positive	Bivariate analysis		Multivariable analysis	
	*N = 2412*	*N = 1396*	OR (95% CI)^*1*^	p-value	aOR (95% CI)^*2*^	p-value
**Age in years, n (%)**						
* ≤ 20*	295	55 (18.6)	Ref		Ref	
* > 20*	2117	1341 (63.3)	7.5 (5.6–10.2)	<0.001	4.6 (3.0 - 7.0)	<0.001
**Sex, n (%)**						
* Male*	1011	678 (67.1)	Ref		Ref	
* Female*	1401	718 (51.4)	0.5 (0.4 - 0.6)	<0.001	0.4 (0.4 - 0.5)	<0.001
**Ethnicity, n (%)**						
* Lao-Loum*	1892	1090 (57.6)	Ref		Ref	
* Non-Lao-Loum*	520	306 (58.9)	1.1 (0.9 - 1.3)	0.613	1.1 (0.8 - 1.5)	0.701
Occupation, n (%) *						
* Student*	231	31 (13.4)	Ref		Ref	
* Business/commerce*	371	190 (51.2)	6.8 (4.4–10.4)	<0.001	2.5 (1.4 - 4.4)	0.001
* Farmer/ Labour worker*	917	597 (65.1)	12.0 (8.1–18.0)	<0.001	2.9 (1.7 - 4.9)	<0.001
* Office staff*	216	148 (68.5)	14.0 (8.7–22.6)	<0.001	3.6 (2.0 - 6.6)	<0.001
* Unemployed*	675	429 (63.6)	11.3 (7.5–17.0)	<0.001	4.1 (2.5 - 7.0)	<0.001
**Province, n (%)**						
* Vientiane capital*	730	315 (43.2)	Ref		Ref	
* Champasack*	414	288 (69.6)	3.0 (2.3–3.9)	<0.001	3.0 (2.3 - 4.0)	<0.001
* Luangprabang*	461	250 (54.2)	1.6 (1.2 - 2.0)	<0.001	1.4 (1.1 - 1.9)	0.01
* Oudomxay*	396	240 (60.6)	2.0 (1.6–2.6)	<0.001	1.7 (1.2 - 2.5)	0.004
* Savannakhet*	411	303 (73.7)	3.7 (2.8–4.8)	<0.001	3.5 (2.6 - 4.7)	<0.001

Age-groups were collated to allow greater power of analysis.

^1^OR, odds ratio; CI, confidence interval.

^2^aOR, adjusted odds ratio; CI, confidence interval.

*Occupation, n = 2410.

### Dual anti-HAV and anti–HEV positivity

Participants aged >20 years had higher double-seropositve prevalence compared to those aged ≤20 years (aOR=14.8, 95% CI = 8.3–26.7, p < 0.001). Females showed statistically lower rates of double positivity compared to males (aOR=0.5, 95% CI = 0.4–0.6, p < 0.001). Students were less likely to be positive for both anti-HAV and anti-HEV compared to participants in other occupations. Additionally, participants from Vientiane capital had significantly lower double-positive seroprevalence than all other provinces (Table 4).

**Table 4:** Bivariate analysis of characteristics by double anti-HAV and anti-HEV seropositivity, (N = 2412).

Characteristics	Overall	Double positivity	Bivariate analysis		Multi variable analysis	
	*N = 2412*	*N = 1289*	OR (95% CI)^*1*^	p-value	aOR (95% CI)^*2*^	p-value
**Age in years, n (%)**						
* ≤ 20 years*	295	15 (5.1)	Ref		Ref	
* > 20 years*	2117	1274 (60.2)	28.2 (17.3 - 49.9)	<0.001	14.8 (8.2–26.7)	<0.001
**Sex, n (%)**						
* Male*	1011	616 (60.9)	Ref		Ref	
* Female*	1401	673 (48.0)	0.59 (0.50 - 0.70)	<0.001	0.5 (0.4–0.6)	<0.001
**Ethnicity, n (%)**						
* Lao-Loum*	1892	999 (52.8)	Ref		Ref	
* Non-Lao-Loum*	520	290 (55.8)	1.13 (0.93 - 1.37)	0.230	1.1 (0.8–1.6)	0.474
Occupation, n (%) *						
* Student*	233	8 (3.4)	Ref		Ref	
* Business/commerce*	371	179 (48.2)	26.2 (13.4 - 59.3)	<0.001	5.7 (2.4–13.6)	<0.001
* Farmer/ Labour worker*	917	551 (60.1)	42.3 (22.1 - 94.5)	<0.001	6.3 (2.7–14.8)	<0.001
* Office staff*	216	143 (66.2)	55.10 (27.3 - 127)	<0.001	8.7 (3.6–21.3)	<0.001
* Unemployed*	673	407 (60.5)	43.0 (22.3 - 96.4)	<0.001	9.9 (4.2–22.9)	<0.001
**Province, n (%)**						
* Vientiane capital*	730	288 (39.5)	Ref		Ref	
* Champasack*	414	263 (63.5)	2.67 (2.09 - 3.43)	<0.001	2.7 (2.1–3.6)	<0.001
* Luangprabang*	461	241 (52.3)	1.68 (1.33 - 2.13)	<0.001	1.6 (1.2–2.1)	0.001
* Oudomxay*	396	227 (57.3)	2.06 (1.61 - 2.65)	<0.001	1.7 (1.2–2.6)	0.004
* Savannakhet*	411	270 (65.7)	2.94 (2.29 - 3.79)	<0.001	2.9 (2.2–3.9)	<0.001

Age-groups were collated to allow greater power of analysis.

^1^OR, odds ratio; CI, confidence interval.

^2^aOR, adjusted odds ratio; CI, confidence interval.

*Occupation, n = 2410.

## Discussion

HAV and HEV are common in low- and middle-income countries where they are often associated with low WASH levels [3,6]. In areas where WASH levels have increased, such as in neighbouring Thailand, there is a concomitant decrease in infections with HAV and HEV, resulting in a shift of the age-stratified seroprevalence curve to the right [14]. In this study, we aimed to determine the seroprevalence of anti-HAV and anti-HEV in the general population in five provinces in Lao PDR, a country with poor WASH levels and no routine HAV or HEV vaccination.

Anti-HAV seroprevalence was 84.3% overall, indicating high levels of exposure in the general population. The seroprevalence increased from 26.8% in age group 20 years or less, to 92.4% in those aged over 20 years, reflecting cumulative exposure and long-term persistence of IgG [2]. In Thailand, a study conducted in 2014 reported a seroprevalence of only 34.5%, with highest prevalence (92.5%) observed among individual over 50 years of age. Decreased seroprevalence over several decades was suggested to reflect improved WASH levels [14]. In Vietnam, seroprevalence was 69.2%, with 57.9% in urban areas and 80.7% in rural areas [15]. These data emphasise the variation in seroprevalence (exposure) to HAV in different settings, reflecting different risk factors such as WASH levels.

In the current study, non-Lao-Loum ethnicity and occupations other than students were positively linked with anti-HAV seropositivity, possibly because other ethnicities are more commonly found in rural areas with difficult access to clean water and students are more likely to be younger and/or perhaps come from households with higher socioeconomic status, linked to improved sanitation and hygiene levels [16,17]. In a smaller, more geographically limited study, we previously documented differences between Xiengkhouang province and Vientiane capital with anti-HAV seroprevalence rates of 62% and 45.5%, respectively [10]. However, the seroprevalence rates documented here are much higher with a range from 77.1% in Vientiane capital to 92.9% in Oudomxay, possibly because of the high mean age of nearly 43 years of the study participants. In the absence of vaccination, our findings suggest a very high HAV exposure rate in many regions of Lao PDR. While part of the differences between provinces might be related to variations in the sanitation and hygiene practices, other factors such as age distribution or ethnicity and occupation profile of the study populations and possibly also previous outbreaks of hepatitis A probably play a role as well. We also found considerable variation between provinces concerning the estimated age at which 50% of the study population are seropositive, with Vientiane Capital and Savannakhet province having the highest age (26 years) and Oudomxay the lowest (12 years), suggesting highest exposure in children from Oudomxay. According to the Lao Social Indicator Survey 2018, the proportion of households with improved WASH level is 81.1% for Vientiane capital, while this rate is only 26.6% for Savannakhet and 27.8% for Oudomxay [17], again suggesting that WASH levels alone may not fully explain the differences in seropositivity observed in our study.

The overall seroprevalence of anti-HEV antibodies was 57.9%, which is similar to the 59.1% we had previously found in cattle farmers from Vientiane capital [12]. As with anti-HAV, the antibodies against HEV are long-lived and therefore seroprevalence increased from age group 18.6% in those aged 20 years old or younger, to 63.3% in those aged more than 20 years. In Vientiane Capital, a seroprevalence of 43.2% was found, which is very similar to the 41.0% among professionals exposed to pigs [11] and the 43.9% among villagers without cattle and pigs [12] but higher than the 18.1% we had detected in urban blood donors from Vientiane capital [11], most likely due to the younger age of the blood donors. Savannakhet had the lowest (22 years) and Vientiane capital the highest (49 years) estimated age at which 50% of the study population are seropositive, suggesting that exposure is lower in children in Vientiane. This could be due to the largely different WASH levels, since contaminated water was suggested to be a major source of HEV infection in Lao PDR [12] and elsewhere [18,19], but also to the high number of smallholder farmers in Savannakhet [20], keeping pigs, cattle and other ruminants potentially infected with HEV [12]. Also unknown differences in study population characteristics and unreported previous outbreaks might have contributed to the observed differences [11,12]. Like for HAV, students demonstrated a lower seroprevalence of anti-HEV, probably for the same reasons. Males, in contrast, were more likely seropositive, possibly because of a higher exposure risk due to more frequent animal contact [21].

Almost all anti-HEV positive participants were also anti-HAV positive (1289/1396; 92.3%), which explains why the characteristics of the double positives largely reflect the findings described for the anti-HEV positive cohort.

Our study has some limitations. Firstly, we used serum samples collected for a cross-sectional anti-SARS-CoV-2 serostudy and thus crucial information on risk factors and details on water source and sanitation are missing. Therefore, we can only conjecture regarding the risk factors involved. However, these are likely to relate to known factors such as swine contact and poor WASH. Secondly, our data only cover five provinces and does therefore not provide a comprehensive overview of the situation across the entire country. It is possible that more rural areas have a higher seroprevalence due to increased risk factors. However, to our knowledge this is the most comprehensive report on anti-HAV and anti-HEV seroprevalence available for Lao PDR to date and clearly highlights a very high exposure of the population.

## Conclusion

Our findings indicate that HAV and HEV exposure is widespread in Lao PDR, with notable differences between provinces. These significant differences according to location and socio-demographics may be a result of variation of exposure to the viruses, such as through WASH-related risks (manuscript in preparation), occupational exposure and animal contact. A One Health approach is warranted, especially in the context of HEV which can be transmitted directly from animals, or indirectly through contaminated water.

Vientiane Capital, which has achieved Open Defecation Free status, demonstrated the lowest seroprevalence of both infections, strongly suggesting the importance of improved WASH infrastructure in reducing HAV and HEV transmission. Notably, although anti-HAV seroprevalence (indicating exposure) was high overall, low levels of exposure in children, particularly from Vientiane, suggest a transition from high to intermediate/low endemicity. Further studies are warranted to determine the utility of childhood HAV vaccination in this population, as well as to identify the key transmission routes of HAV and HEV in Lao PDR, particularly the importance of low standard WASH levels.

## Supporting information

S1 Data data_PLOS ONE. (XLSX)

## Abbreviations

CI: confidence intervals
ELISA: enzyme-linked immunosorbent assay
HAV: hepatitis A virus
HEV: hepatitis E virus
NECHR: national ethics committee for health research
Lao PDR: Lao People’s democratic republic
WASH: water, sanitation and hygiene
WHO: World Health Organisation.
